# Experimentation and Numerical Modeling of Peak Temperature in the Weld Joint during Rotary Friction Welding of Dissimilar Plastic Rods

**DOI:** 10.3390/polym15092124

**Published:** 2023-04-29

**Authors:** Chil-Chyuan Kuo, Naruboyana Gurumurthy, Hong-Wei Chen, Song-Hua Hunag

**Affiliations:** 1 Department of Mechanical Engineering, Ming Chi University of Technology, No. 84, Gungjuan Road, New Taipei City 243, Taiwan; 2 Research Center for Intelligent Medical Devices, Ming Chi University of Technology, No. 84, Gungjuan Road, New Taipei City 243, Taiwan; 3 Department of Mechanical Engineering, Chang Gung University, No. 259, Wenhua 1st Road, Guishan District, Taoyuan City 33302, Taiwan; 4 Department of Mechanical Engineering, Presidency University, Rajankunte, Near Yelhanka, Bangalore 700073, India; 5 Li-Yin Technology Co., Ltd., No. 37, Lane 151, Section 1, Zhongxing Road, Wugu District, New Taipei City 241, Taiwan

**Keywords:** energy consumption, environmental effects, rotary friction welding, acrylonitrile butadiene styrene, polycarbonate, peak temperature, weld joint, environmental pollution

## Abstract

Rotary friction welding (RFW) could result in lower welding temperature, energy consumption, or environmental effects as compared with fusion welding processes. RFW is a green manufacturing technology with little environmental pollution in the field of joining methods. Thus, RFW is widely employed to manufacture green products. In general, the welding quality of welded parts, such as tensile strength, bending strength, and surface hardness is affected by the peak temperature in the weld joint during the RFW of dissimilar plastic rods. However, hitherto little is known about the domain knowledge of RFW of acrylonitrile butadiene styrene (ABS) and polycarbonate (PC) polymer rods. To prevent random efforts and energy consumption, a green method to predict the peak temperature in the weld joint of dissimilar RFW of ABS and PC rods was proposed. The main objective of this work is to investigate the peak temperature in the weld joint during the RFW using COMSOL multiphysics software for establishing an empirical technical database of RFW of dissimilar polymer rods under different rotational speeds. The main findings include that the peak temperature affecting the mechanical properties of RFW of PC and ABS can be determined by the simulation model proposed in this work. The average error of predicting the peak temperature using COMSOL software for five different rotational speeds is about 15 °C. The mesh element count of 875,688 is the optimal number of meshes for predicting peak temperature in the weld joint. The bending strength of the welded part (y) using peak welding temperature (x) can be predicted by the equation of y = −0.019 x^2^ + 5.081x − 200.75 with a correlation coefficient of 0.8857. The average shore A surface hardness, impact energy, and bending strength of the welded parts were found to be increased with increasing the rotational speed of RFW.

## 1. Introduction

The energy consumption associated with the pre-processing, welding process, and post-processing steps of rotary friction welding (RFW) is obviously lower than gas metal arc welding. The advantage of adhesive bonding is that it can be bonded with dissimilar materials economically [1,2]. However, it is not suitable for industrial applications because of its low working efficiency. The RFW [3,4,5,6,7] is a solid-state joining process using a compressive axial force. According to practical experience in the industry, the RFW gives many advantages. The welding process has a lower peak temperature in the weld joint than fusion welding (FW) [8,9]. Thus, intermetallic formation can be reduced. In addition, a wide range of dissimilar materials can be joined. The welding process does not need a filler metal and shielding gas. Many common defects associated with high-temperature melting and solidification during FW, such as solidification cracks or pores can be avoided. Therefore, welded parts with low defect rates as well as low distortion can be obtained easily. The material used and manufacturing costs are reduced greatly compared with subtractive techniques, such as machining from buck materials [10,11].

Zhang et al. [12] proposed a thermal compression bonding process to influence the friction flow of formation intermetallic compounds in friction welding. Results revealed that the frictional flow significantly promoted the formation of sub-micron-sized intermetallic compounds along the weld interface. Ma et al. [13] studied the effects of temperature on the mechanical performances of friction stir welded aluminum (Al) alloy joints. The results revealed that the pin increases heat input and material flow at the bottom, reducing the gradient along the thickness. Eliseev et al. [14] focused on the structural evolution in the transfer layer of Al alloy welds fabricated with various axial loads. It was found that the volume fraction and size of incoherent intermetallic particles decrease towards the center of the layer. Iftikhar et al. [15] investigate the friction stir spot welding of thermoplastic polymers. The results revealed that it is difficult to optimize the welding process parameters because of the dependence on many factors. Pereira et al. [16] focused on the influence of different welding methods on the mechanical strength of friction stir welds of thermoplastic polymers. The results revealed that the increase in the welding speed ratio increased the joint efficiency. Unfortunately, it was difficult to establish mathematical models because of the variation in welding conditions. Wang et al. [17] studied RFW on dissimilar brass bars using a pre-heating approach. It was found that a very narrow intermetallic compound layer was formed. Ishraq et al. [18] analyzed the weld strength by optimizing the process parameters of RFW at different levels. It was found that the reason for the high strength of a selected material is the optimal level of fiberglass. Hangai et al. [19] investigated the effects of the porosity of Al foam on the RFW. It was found that the Al foam can be welded to a polycarbonate plate by RFW. Dhooge et al. [20] proposed a promising welding approach for the fully automatic joining of pipelines, which is a new variant of the conventional friction welding process, and discussed the optimization of the duration of the friction phase.

Polymer is frequently used in some structures, such as automobiles, pressure vessels, and aircraft. Especially, the major difference between metal and polymer is that polymer is more lightweight and anti-corrosive than metal. Polycarbonate (PC) and acrylonitrile butadiene styrene (ABS) are compatible with each other since they have similar polarities. Therefore, mixtures of ABS and PC have been widely employed in engineering applications. In general, the major shortcomings of the trial-and-error method involve random efforts and energy consumption. It is well known that the [21,22,23,24,25] thermal analysis solutions can solve the most complex thermal challenges to predict the temperature. Unfortunately, hitherto little is known about the domain knowledge of the RFW of ABS and PC polymer rods. For this reason, the main objective of this study is to establish domain knowledge of the RFW of ABS and PC rods. To prevent random efforts and energy consumption, a green method to predict peak temperature in the weld joint of dissimilar RFW of ABS and PC rods was proposed. Therefore, the COMSOL multiphysics software [26] was employed to investigate the peak temperature in the weld joint during dissimilar RFW of ABS and PC rods. An infrared thermal imager was employed to investigate the peak temperature in the weld joint during RFW under five different rotational speeds. After FRW, the peak temperature obtained by the experiment was compared with the simulation results. Finally, an empirical technical database of RFW of dissimilar polymer rods under different rotational speeds is established.

## 2. Experimental Details

**Figure 1:**
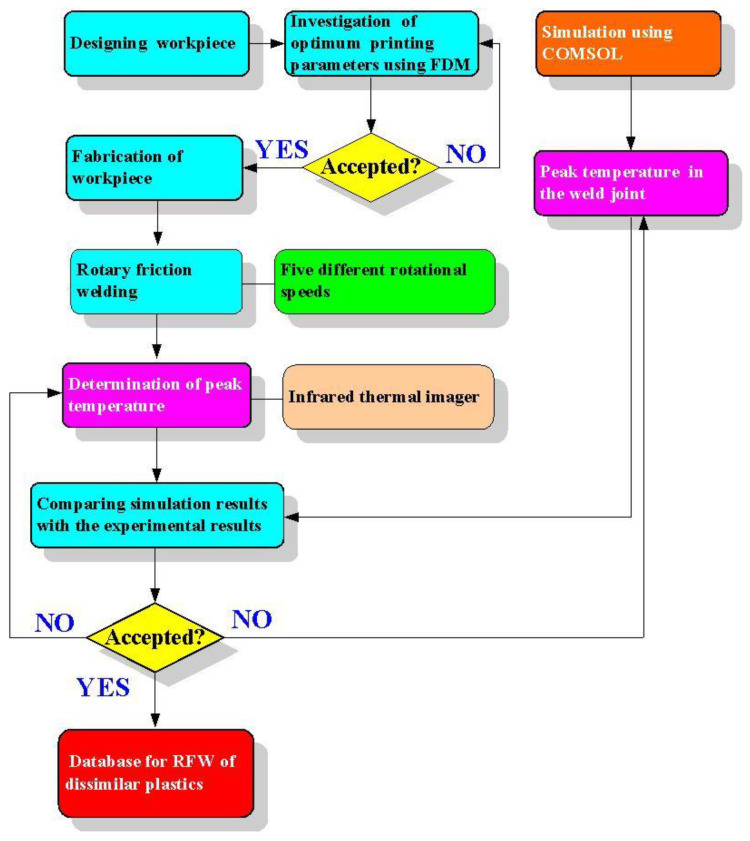
Research flowchart of this study.

Figure 1 shows the research flowchart of this study. The research flow involves designing the workpiece, investigating optimum printing parameters using fused deposition modeling (FDM) [27], fabricating the workpieces, FRW, the determination of peak temperature by an infrared thermal imager [28] under five different rotational speeds [29], investigating the peak temperature by applying COMSOL multiphysics software, comparing the simulation results with the experimental results, and proposing a database of dissimilar RFW of ABS and PC rods. In the COMSOL multiphysics software, an attempt was made to simulate the peak temperature in the weld joint under the cycle time of 60 s. Generally, ABS thermoplastic material has good toughness and impact properties [30]. PC is an engineering thermoplastic material that has durability and thermal insulation [31,32]. In this study, two different kinds of filaments, i.e., ABS (Thunder 3D Inc., New Taipei City, Taiwan) and PC (Thunder 3D Inc., New Taipei City, Taiwan) were used to print welding workpieces.

**Figure 2:**
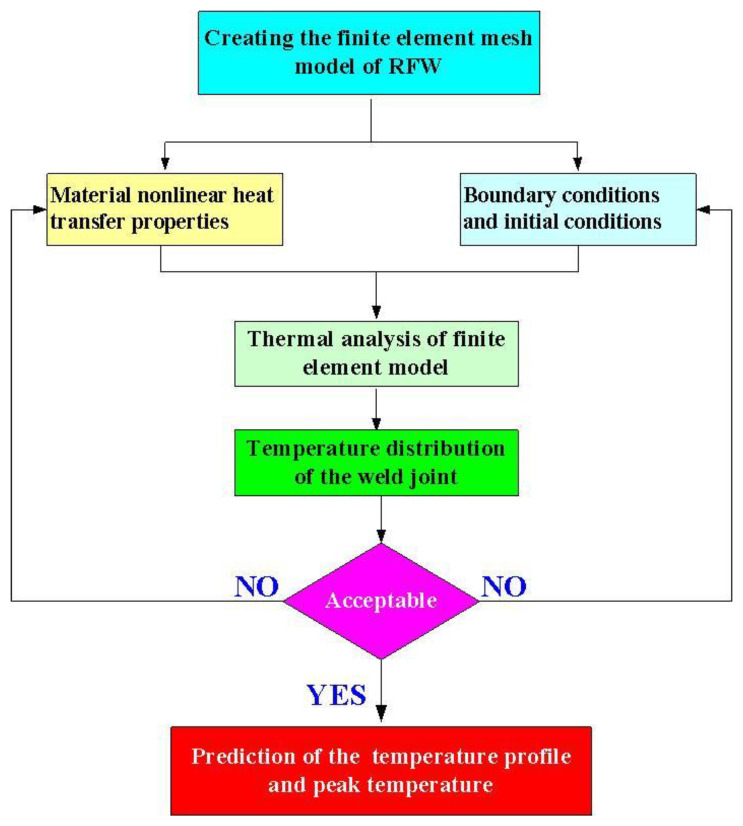
Flowchart of numerical simulation by applying COMSOL multiphysics software.

Figure 2 shows the flowchart of numerical simulation by applying COMSOL multiphysics software, which includes the thermal pattern analysis and the suitable boundary conditions for the RFW model. The entire process involves establishing the finite element mesh model of RFW, setting the parameters for RFW, setting the material nonlinear heat transfer properties, setting both boundary conditions and initial conditions, thermal analysis of the finite element model, analysis of the temperature distribution of the weld joint, analysis of the temperature rise in the weld bead, and the prediction of the temperature profile and peak temperature. The welding workpiece is a cylindrical rod with a diameter of 20 mm and a length of 40 mm. The UltiMaker Cura software was then employed to generate a printing program. The welding specimens were built with a fused deposition modeling (FDM) machine [33,34]. The build direction of the printed welding specimen was determined according to fewer supports, high dimensional accuracy, less printing time, and high surface quality. Firstly, the welding workpiece was designed using software named Cero (parametric technology corporation Inc., New Taipei City, Taiwan). According to practical experience, the printing parameters for PC welding workpieces involve a printing temperature of 245 °C, a printing speed of 50 mm/s, a layer thickness of 0.1 mm, and a printing bed temperature of 100 °C. In addition, the printing parameters for ABS welding workpieces involve a printing temperature of 230 °C, a printing speed of 45 mm/s, a layer thickness of 0.1 mm, and a printing bed temperature of 100 °C.

A conventional lathe was used to perform RFW. In general, the RFW provides axial movement to obtain the required weld strength. During RFW, one welding specimen was rotated at a constant speed while the other was held stationary. Two welding specimens were brought together under pressure for a certain period of time. In this study, the cycle time of FW was set to 60 s. The cycle time involves a friction time of 30 s, a weld time of 20 s, and a cooling time of 10 s. The burn-off length was set to 2 mm since the FW was carried out 20 times with a weld length of 0.1 mm each time. The selection of five rotational speeds is mainly based on the specifications of the lathe used in this study. To investigate the effects of rotational speed on peak temperature in the weld joint, five different rotational speeds, i.e., 330 rpm, 490 rpm, 650 rpm, 950 rpm, and 1350 rpm were carried out in this study. The peak temperature in the weld joint during the RFW of dissimilar specimens was monitored and recorded using an infrared camera (BI-TM-F01P, Panrico trading Inc., New Taipei City, Taiwan). After RFW, the shore A surface hardness test (MET-HG-A, SEAT Inc. New Taipei City, Taiwan), three-point bending test (RH-30, Shimadzu Inc., Kyoto, Japan), and impact test (780, Instron Inc., Massachusetts, MA, USA) were carried out to evaluate the mechanical properties of the frictionally welded parts.

## 3. Results and Discussion

**Figure 3:**
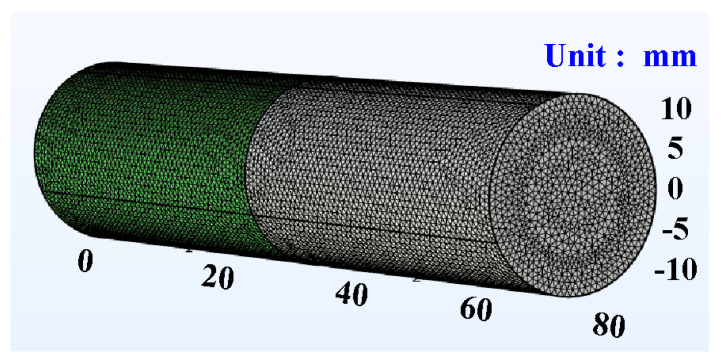
Geometry and mesh of the workpieces in the simulation for the element size of 0.8 mm.

**Figure 4:**
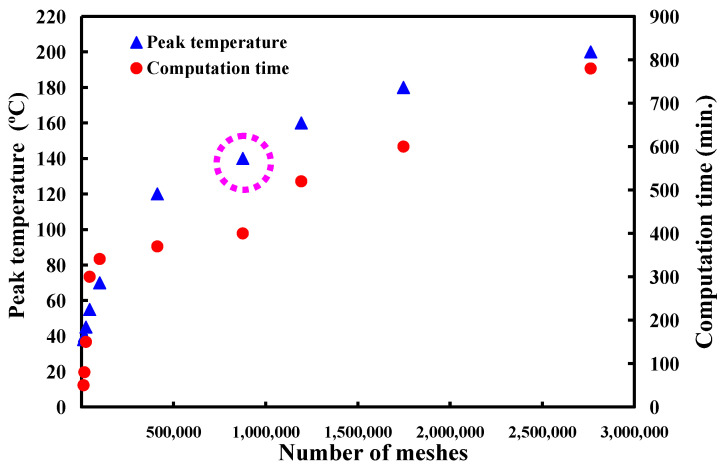
Number of meshes as a function of computing time and peak temperature in the weld joint.

**Figure 5:**
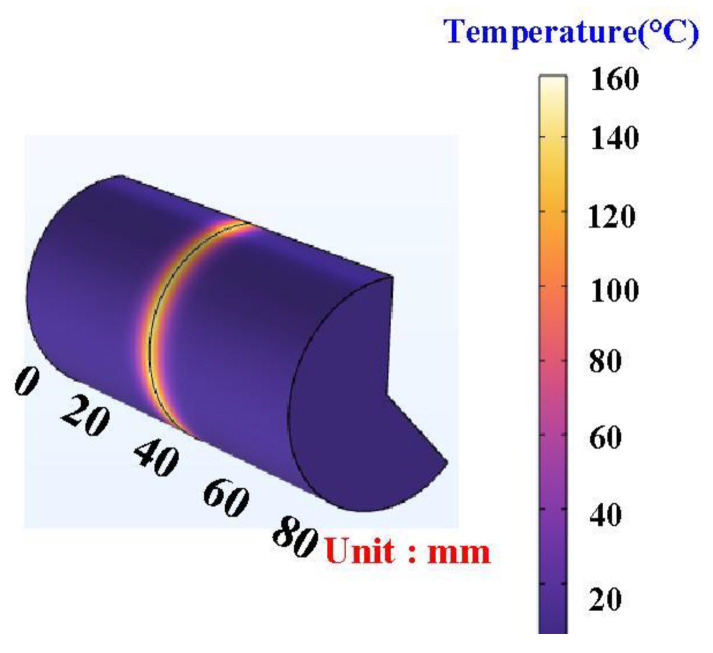
Temperature distributions for RFW of two dissimilar workpieces.

**Figure 6:**
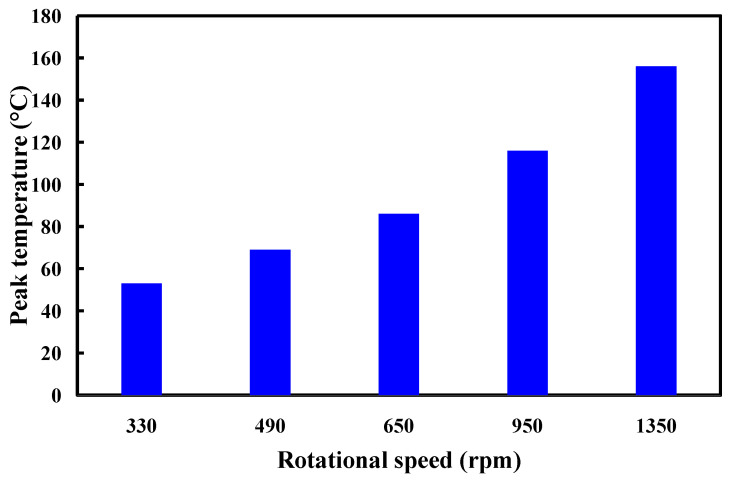
Peak temperature in the weld joint for five diffident rotational speeds predicted by COMSOL multiphysics software.

In general, the welding quality of the welded parts was influenced by the peak temperature in the weld joint during RFW of dissimilar plastic rods since the mechanical properties were affected by the peak temperature during RFW [35]. In this study, the COMSOL multiphysics software was used to investigate the peak temperature in the weld joint. Ten different kinds of element sizes, i.e., 0.4 mm, 0.5 mm, 0.6 mm, 0.7 mm, 0.8 mm, 0.9 mm, 1.0 mm, 1.1 mm, 1.2 mm, and 1.3 mm were performed to investigate the peak temperature in the weld joint during RFW. Figure 3 shows the geometry and mesh of the workpieces in the simulation. The element size of the processed workpieces is 0.8 mm. To determine the type of mesh suitable for RFW, Figure 4 describes the number of meshes as a function of computing time and peak temperature in the weld joint. As can be seen, a higher number of meshes has a higher computation time. In addition, calculating the peak temperature in the weld joint during RFW of dissimilar plastic rods by applying COMSOL multiphysics software was feasible [36]. Especially, the peak temperature predicted by the COMSOL multiphysics software using a mesh element count of 875,688 is very close to that obtained by the experimental result. This means that the mesh element count of 875,688 seems to be the optimal number of meshes for predicting peak temperature in the weld joint. Figure 5 shows the temperature distributions for RFW of two dissimilar workpieces. The thermal model takes into account various parameters such as friction pressure, rotational speed, feed rate, and material properties of the polymer rods being joined. As can be seen, the temperature distribution, amount of energy required to achieve a successful weld, and heat-affected zone can be calculated based on the thermal model in the COMSOL multiphysics software. Figure 6 shows the peak temperature in the weld joint for five diffident rotational speeds predicted by COMSOL multiphysics software. The peak temperatures in the weld joint for five diffident rotational speeds of 330 rpm, 490 rpm, 650 rpm, 950rpm, and 1350 rpm are 53 °C, 70 °C, 86 °C,116 °C, and 156 °C, respectively.

**Figure 7:**
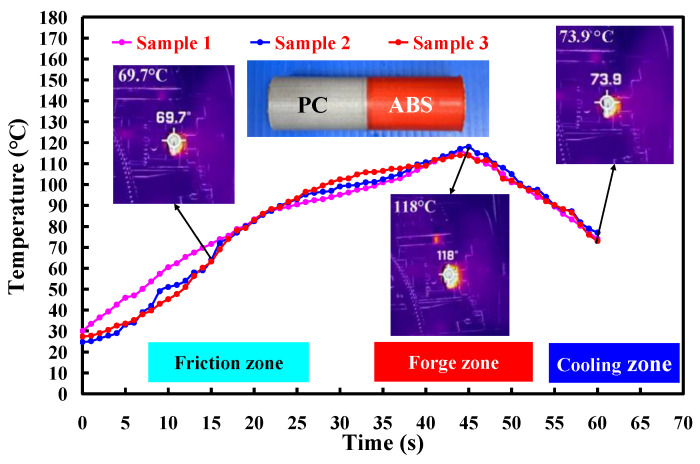
Temperature history in the weld joint for RFW of PC and ABS rods at a rotational speed of 950 rpm.

**Figure 8:**
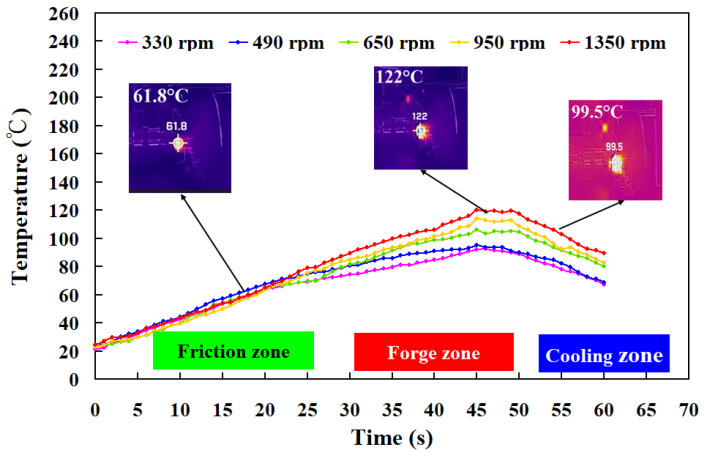
Temperature history in the weld joint for RFW of PC and ABS rods at five different rotational speeds.

To investigate the repeatability of the RFW experiments in this study, three samples were used in this study. The material emissivity of ABS and PC is about 0.92 and 0.95, respectively. The image resolution for the thermal imaging data is about 1440 × 1080 pixels. In general, the melting temperature for PC and ABS is about 155 ± 10 °C and 245 ± 10 °C, respectively [37,38]. Figure 7 shows the relationship between weld joint temperature and FW time for PLA and PLA rods at a rotational speed of 950 rpm. As can be seen, the relationship between weld time and joint temperature for the RFW of ABS and PC workpieces at a rotational speed of 950 rpm is repeatable. The peak temperature in the weld joint was found to be approximately 118 °C. This result is supported by the experiment proposed by Mura et al. [39], showing the glass transition temperature of PC-ABS is approximately 125 °C. Figure 8 shows the relationship between weld joint temperature and FW time for PLA and PLA rods at five different rotational speeds. The results showed that the average peak temperatures of weld joint for rotational speeds of 330 rpm, 490 rpm, 650 rpm, 950 rpm, and 1350 rpm are approximately 88 °C, 99 °C, 106 °C, 114 °C, and 153 °C, respectively. The results revealed that the peak temperatures in the weld joint increase gradually with increasing rotational speed.

**Figure 9:**
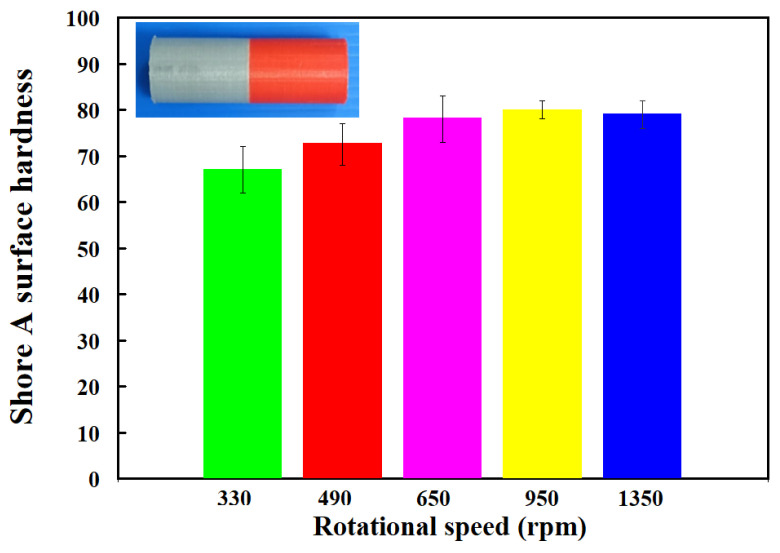
Surface hardness in the weld joint for RFW of PC and ABS rods at five different rotational speeds.

**Figure 10:**
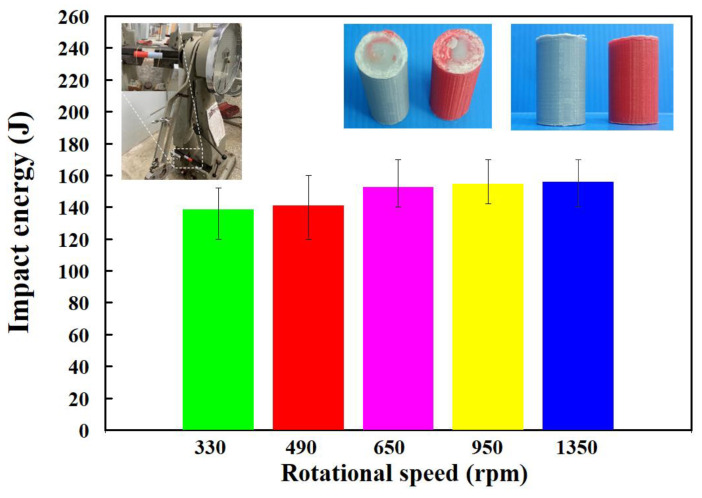
Impact energy of dissimilar polymer rods welded by five different rotational speeds.

**Figure 11:**
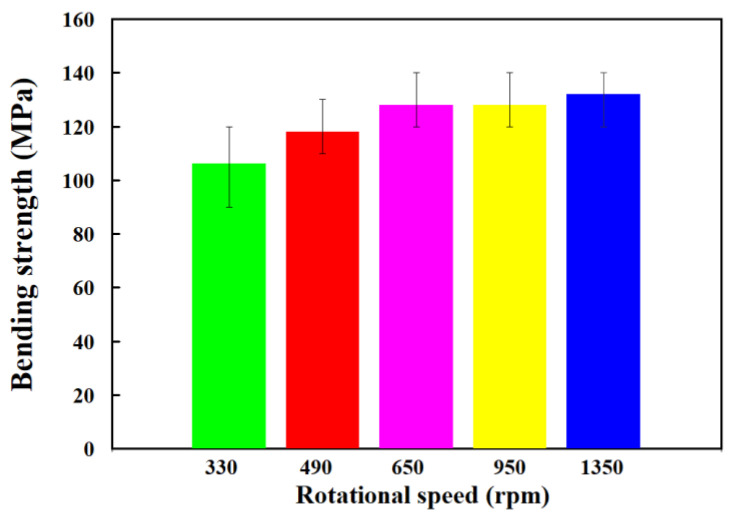
Bending strength of the welded part under five different rotational speeds.

**Figure 12:**
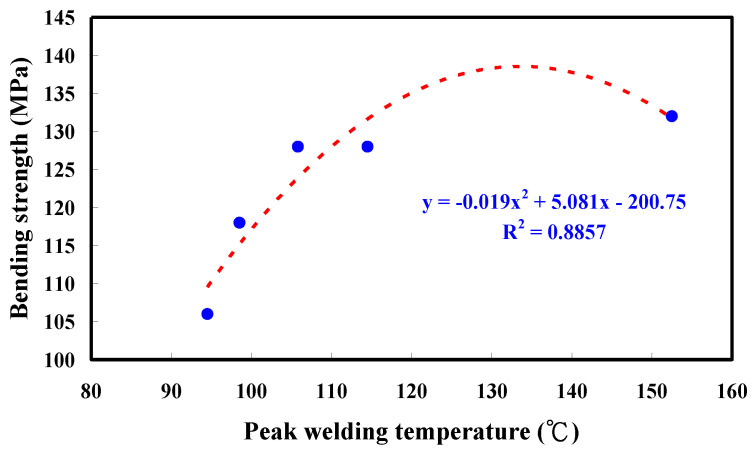
Bending strength as a function of peak welding temperature.

Figure 9 shows the surface hardness in the weld joint for RFW of PC and ABS rods at five different rotational speeds. As can be seen, the average shore A surface hardness in the weld joint is increased with increasing the rotational speed of RFW. Figure 10 shows the impact energy of dissimilar polymer rods welded at five different rotational speeds. The results showed that the impact energy in the weld joint is increased with increasing the rotational speed of RFW. This result was also confirmed by the experiment proposed by Dhaiwat et al. [40]. Figure 11 shows the ending strength of the welded part under five different rotational speeds. As can be seen, the bending strength in the weld joint is increased with increasing the rotational speed of RFW. Figure 12 shows the bending strength as a function of peak welding temperature. It was found that the equation of y = −0.019 x^2^ + 5.081x − 200.75 with the correlation coefficient [41] of 0.8857 seems to be an optimum trend equation for predicting the bending strength of the welded part (y) using peak welding temperature (x).

**Figure 13:**
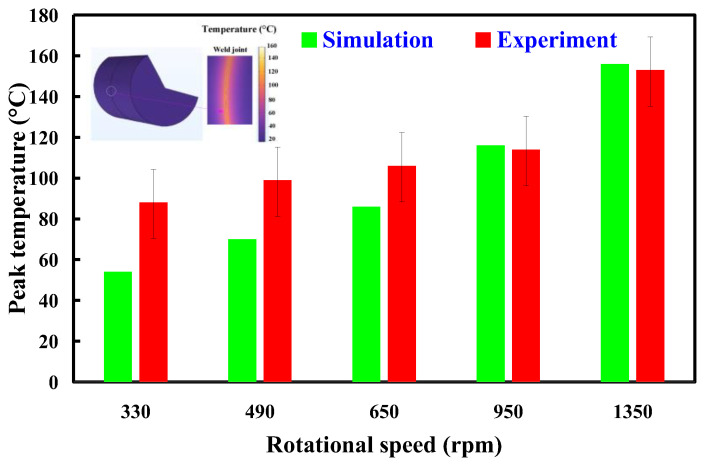
Comparison of the numerical simulation and experimental results of the peak temperature for RFW of PC and ABS rods at five different rotational speeds.

**Figure 14:**
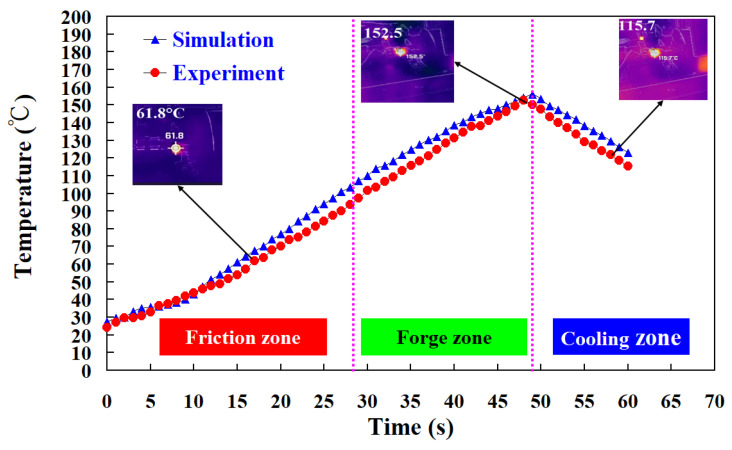
Comparison of the numerical simulation and experimental results of the temperature profile.

Figure 13 shows the comparison of the numerical simulation and experimental results of the peak temperature for RFW of PC and ABS rods at five different rotational speeds. Figure 14 shows the comparison of the numerical simulation and experimental results of the temperature profile. As can be seen, the difference in the peak temperature between simulation and experimental results for rotational speeds of 330 rpm, 490 rpm, 650 rpm, 950 rpm, and 1350 rpm is about 34 °C, 29 °C, 20 °C, −2 °C, and −3 °C, respectively. Thus, the average error of predicting the peak temperature by applying COMSOL software for five different rotational speeds is about 15 °C.

In practice, one of the advantages of the FRW is reduced energy consumption as compared to arc welding processes [42]. Therefore, the RFW of dissimilar polymer rods is a green manufacturing technique for joining dissimilar polymer rods and meets sustainable development (SDG_S_ 9 and 12) [43]. In general, this technique can be used for jointing automotive components, aircraft components, axle shafts, aerospace components, fluid mechanical components, or transmission shafts [44,45]. In this study, a conventional lathe was employed to perform RFW of ABS and PC rods. In future investigations, the computer numerical control turning machine [46] is recommended to perform RFW of ABS and PC rods since the feed rate of RFW can be precisely controlled to replace human error. In addition, the rotational speed [47,48,49,50,51,52] can be changed during the whole process of RFW. These topics are interesting research topics and are currently being investigated, and the results will be presented in later works.

## 4. Conclusions

An energy-related key performance indicator is frequently used as a tool to evaluate the energy consumption of manufacturing processes, focusing on energy consumption and environmental impact. RFW is a green manufacturing process and is becoming useful in lots of industrial applications. RFW is a sustainable manufacturing technology with low energy consumption since it generates heat through mechanical friction between thermoplastics. The advantages of RFW include being free from thermal distortion or porosity which are defects seen in other welding techniques. In this work, an analysis of the peak temperature in the weld joint during dissimilar RFW of ABS and PC rods by applying the COMSOL multiphysics software is presented. The main conclusions from the experimental work in this study are as follows:

1. The use of COMSOL software was feasible for calculating the peak temperature in the weld joint during dissimilar RFW of ABS and PC rods. The mesh element count of 875,688 is the optimal number of meshes for predicting peak temperature in the weld joint. The average error of predicting the peak temperature using the COMSOL software for five different rotational speeds is about 15 °C.
2. The bending strength of the welded part (y) using peak welding temperature (x) can be predicted by the equation of y = −0.019 x^2^ + 5.081x − 200.75 with the correlation coefficient with a correlation coefficient of 0.8857.
3. The bending strength, average shore A surface hardness, and impact energy of the welded parts were increased with increasing the rotational speed of RFW.

## Data Availability

Data and materials are available.
